# Patients’ views on their decision making during inpatient rehabilitation after newly acquired spinal cord injury—A qualitative interview‐based study

**DOI:** 10.1111/hex.12559

**Published:** 2017-03-24

**Authors:** Anke Scheel‐Sailer, Marcel W. Post, Franz Michel, Tatjana Weidmann‐Hügle, Ruth Baumann Hölzle

**Affiliations:** ^1^ Swiss Paraplegic Centre Nottwil Switzerland; ^2^ Swiss Paraplegic Research Nottwil Switzerland; ^3^ University of Groningen University Medical Center Groningen Department of Rehabilitation Medicine Groningen the Netherlands; ^4^ Center of Excellence in Rehabilitation Medicine Brain Center Rudolf Magnus University Medical Center Utrecht and De Hoogstraat Rehabilitation Utrecht the Netherlands; ^5^ Institute Dialog Ethik Zurich Switzerland; ^6^ Institute of Biomedical Ethics and History of Medicine University of Zurich Zurich Switzerland

**Keywords:** clinical ethics, health communication, legal obligation, personal autonomy, shared decision‐making, subacute care

## Abstract

**Introduction:**

Involving patients in decision making is a legal requirement in many countries, associated with better rehabilitation outcomes, but not easily accomplished during initial inpatient rehabilitation after severe trauma. Providing medical treatment according to the principles of shared decision making is challenging as a point in case for persons with spinal cord injury (SCI).

**Objectives:**

The aim of this study was to retrospectively explore the patients’ views on their participation in decision making during their first inpatient rehabilitation after onset of SCI, in order to optimize treatment concepts.

**Methods:**

A total of 22 participants with SCI were interviewed in‐depth using a semi‐structured interview scheme between 6 months and 35 years post‐onset. Interviews were transcribed verbatim and analysed with the Mayring method for qualitative content analysis.

**Results:**

Participants experienced a substantially reduced ability to participate in decision making during the early phase after SCI. They perceived physical, psychological and environmental factors to have impacted upon this ability. Patients mentioned regaining their ability to make decisions was an important goal during their first rehabilitation. Receiving adequate information in an understandable and personalized way was a prerequisite to achieve this goal. Other important factors included medical and psychological condition, personal engagement, time and dialogue with peers.

**Conclusion:**

During the initial rehabilitation of patients with SCI, professionals need to deal with the discrepancy between the obligation to respect a patient's autonomy and their diminished ability for decision making.

## 1 INTRODUCTION

As a normative concept, autonomy is a person's right to be respected as a subject and entails the ability to judge, decide and act upon one's personal attitude, values and reasoning.^1^, ^2^ It is based on the normative concept of human dignity and independent of any personal characteristics or attributes.^3^ Like in various other countries, in 2013 Switzerland introduced new legislation to protect the patient's rights to autonomy and self‐determination.^4^, ^5^, ^6^, ^7^ This right also encourages patient's individuality and a personally established way of living. Shared decision making may be the way to respect and deal with this legal requirement in clinical practice. It refers to a style of communication between a patient and clinician which gives patients’ preferences and values importance.^8^, ^9^ Co‐operative autonomy is based on shared decision making with respect to the special medical condition.^10^, ^11^

Yet, involving patients in decision‐making challenges the patients’ as well as the professionals’ understanding of their respective roles.^12^ Instead of acting as a paternalistic proxy for patients with limited or absent decision‐making ability, professionals are required to act as partners in a shared decision‐making process with the patient having the ultimate decision concerning medical treatment.^13^ Professionals therefore not only require knowledge of the legal framework and ethical issues, but also need to develop new approaches to provide information in an adequate way and be engaged in patient‐focused communication.^14^, ^15^, ^16^, ^17^ Even though the law seems to set a clear framework within which professionals and autonomous patients share treatment decisions, the clinical reality in acute or chronic care settings is often less distinct. Contributing factors to this ambiguity include physicians’ established attitudes towards patient autonomy, lack of time, the knowledge gap between professionals and patients, and the often diminished capacity for self‐determination and decision making of the patient.^1^, ^13^, ^18^, ^19^, ^20^, ^21^, ^22^, ^23^ All these factors and circumstances can lead to tensions between legal requirements and actual clinical practice.

Future implementation efforts to improve patient participation in decision making should address patient‐reported factors together with known clinician‐reported barriers and the wider organizational context.^13^

Spinal cord injury (SCI) is an example of a complex health condition with a life‐threatening acute phase, an individualized comprehensive rehabilitation period and a lifelong chronic phase in which many persons with SCI need long‐term care and are at risk for multiple complications.^24^, ^25^ To our knowledge, only some studies have been published on the participation in decision making by patients with SCI during their first rehabilitation after the onset of their condition.^26^, ^27^ Accordingly, the aim of this study was, first, to explore patient retrospective views on decision making during the initial inpatient rehabilitation and, second, to identify barriers and facilitators for participation in decision making.

## 2 MATERIALS AND METHODS

A qualitative methodology was chosen to gain insight into the complex field of decision making of patients with SCI. The intention was to understand subject‐orientated perspectives and to discover the different perspectives in order to develop generalizable contents and theories, using a structured procedure of interpretation and conclusion.^28^

### 2.1 Patients and setting

Twenty‐two subjects with traumatic SCI without serious cognitive or psychiatric disorder or progressive diseases were selected by purposive sampling, stratified for completeness and level of injury, years since injury, age and gender. Interviews were conducted at the rehabilitation clinic or in the patient's private environment.

### 2.2 Procedures

To develop the topic list for the interviews, a psychologist and a clinical ethicist conducted four focus group interviews with experienced physicians, nurses, physiotherapists, occupational therapists, social workers and a peer counsellor on the decision‐making process. Resting upon the results of these interviews, a topic list for semi‐structured interviews was developed (Table 1). The topics addressed general, organizational and personal aspects of decision making. The questionnaire was tested in a pilot study with four patients and was adjusted subsequently. After obtaining participants’ informed consent, a psychologist and a clinical ethicist without any clinical relationship to the participants conducted all interviews, which were audiotaped and lasted between 45 and 90 minutes.

**Table 1:** Semi‐structured Interview Guideline

*Main topics and the process of decision‐making*
After the onset of an spinal cord injury (SCI), decisions have to be made. Could you share some of these with us?
How did you manage these decisions? How did you decide?
Did other persons participate in your decision making process? Who supported you most?
Did the engagement of other people help you or did it somehow put pressure on you?
Which factors influenced your decision‐making? Money, well‐being, quality of life, etc.?
*Important factors from the institution?*
Did you experience the institution to influence your decision‐making? What or who was helpful or a hindrance?
Did you receive enough information? Did you have enough time to decide?
Could you express your needs, wishes or doubts?
Did you miss anything for a well thought trough decision?
*Perception of the decision‐making process*
Did you have the feeling, that you decided yourself?
Do you think, that having an SCI influences the capacity for decision‐making?
When did you start to decide again?
What do you think helps to regain the capacity for decision‐making?
*Personal attitude regards decision‐making*
How do you decide normally?
How do you decide in a very important situation?
What means “good” or “bad” for you in the decision‐making process? What is your landmark?
How do you realize that your decision was made in the right way?

### 2.3 Descriptive and content analyses

**Figure 1:**
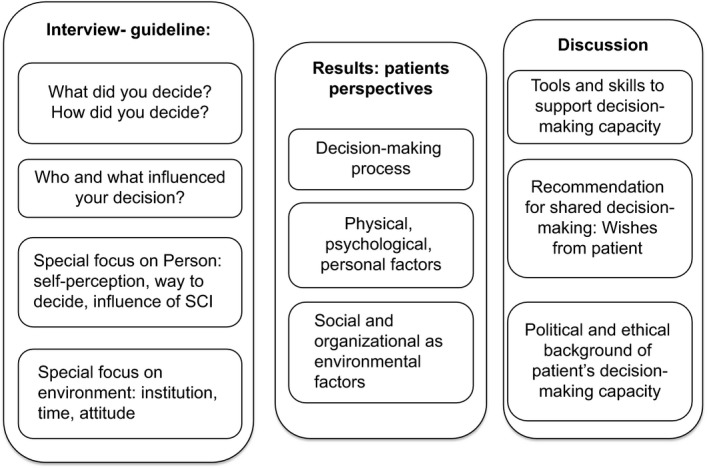
Structure of qualitative analyses from interview to results and discussion in the decision‐making process

After anonymized verbatim interview transcription, a psychologist performed the qualitative content analysis according to Mayring.^28^ The first step was to summarize the interviews in a descriptive way and to mark text fragments related to decision making. Categories were built and text fragments were linked to relevant categories using the data analysis program ATLAS.ti (ATLAS.ti Scientific Software Development GmbH, Berlin, Germany). After revision of these analyses by a second researcher, thematic groups of categories were created. During the qualitative analysis, the structure of the publication appeared, based on interview questions, results and discussion (Figure 1). The results were interpreted and discussed with clinical physicians, psychologists and ethicists.

### 2.4 Statement of Ethics

We certify that all applicable institutional and governmental regulations concerning the ethical use of human volunteers were followed during the course of this research. The study protocol was approved by the Cantonal ethics committee of Lucerne (Registration Number 630).

## 3 RESULTS

Characteristics of the 22 participants are displayed in Table 2. Table 3 summarizes patient‐reported barriers and facilitators in the decision‐making process with respect to the decision‐making process, physical, psychological and personal aspects, attitude from health professionals and organizational aspects.

**Table 2:** Characteristics of participants with spinal cord injury

Characteristic	n	%
Gender		
Female	8	28.5
Male	14	71.5
Level of lesion		
Tetraplegia	8	28.5
Paraplegia	14	71.5
Age when interviewed		
19‐29 years	6	27.7
30‐39 years	2	9.1
40‐49 years	5	22.7
50‐59 years	5	22.7
60‐69 years	4	18.2
Years since lesion		
0‐1 year	7	31.8
2‐4 years	6	27.7
5‐9 years	3	13.6
10‐19 years	2	9.1
20‐40 years	4	18.2
Time since first rehabilitation		
End of first rehabilitation	6	27.2
1‐2.5 years after the first rehabilitation	7	31.8
>5 years after the first rehabilitation	9	40.0

**Table 3:** Barriers and facilitators in the decision‐making processes

Influencing factors	Barriers	Facilitators
Decision‐making process	Personal feelings and health condition	Recovery and activity The commitment for the new life, an active and engaging attitude
Physical, psychological and personal aspects	Shock, pain, medication, reduced health and energy Regress or stagnancy	Well‐being, recovery Acceptance of the new situation Appearance and aesthetics A good body perception and image Personal ideas and engagement Higher independency
Attitude from health professionals	Lack of information about the specific health condition, diagnosis, prognosis, examination and changes in medicationProviding insufficient information about diagnoses, pain and sexualityLack of interest, compassion, being authoritative	A trustful environmentA supporting therapeutic team Detailed information about medicationA respectful and experienced therapeutic teamPronouncing an attractive goal together, higher motivation
Organizational aspect	Rigid structures and limited ideas in the therapeutic teamTime pressure and stress	A friendly and kind atmosphereThe offer of supplementary therapies such as psychotherapy, Feldenkrais, art or music therapy, language educationAn appropriate length of stay in the hospital
Environmental aspects	Separation and divorcePressure from health‐care insurance	Support of peers, family and friendsThe friendly architecture, the spacey rooms, the nature around the institution and the view on the lake and the mountainsEnough space, automatic doors and adapted bathrooms
Aspects of time	A strictly standardized rehabilitation programmeLack of time to reflectUnrealistic goal setting	An individualized rehabilitation scheduleA relevant and intense preparation of the discharge

### 3.1 Decision‐making process

Participants highlighted the onset of SCI as a turning point in their life, characterizing their life following SCI as a new life or a new beginning. During the initial phase of rehabilitation, they experienced difficulties making decisions on even simple activities such as eating by themselves or leaving the ward.

Yes, but then, when I took the first decision myself, it may have begun with trying to feed myself… as a quadriplegic…, you have an ordinary life, and then you are suddenly thrown out of it—and then, you cannot move anything, … you are completely dependent on others, so you give up all responsibility…. In the beginning, it takes a kick in the butt by experienced therapists, … [who have to say] this is it now, you have to feed yourself. At the beginning, you have almost a feeling of rage towards them… and then you begin to make more decisions for yourself and you realize, all the time others decided for you, and this depends completely on me. It takes time to realize this again, though.

Initial acute phase:

I remember that at the beginning, … I wanted to defend myself, but at that time I just wasn't competent enough, so I simply couldn't… I didn't say: ‘I don't want that….’.

I was no longer really myself, you know, after the accident. That's, say, the second half, if you halved the nine months, it has started again. But as in the first half, yes, I felt already less than – simply a beetle on its back.

In general:

As long as I am reasonably ‘together’ and have control over my senses, I want to decide for myself what happens to me.

Many participants preferred not to be forced to decide too many things initially, for example, “when they can be mobilized into the wheel chair,” “how often they should be turned in the bed as pressure prevention” and “how to perform the transfer.” Dealing with SCI was a completely new and challenging experience, resulting in much uncertainty about their future lives. They reported to be initially entirely dependent on experienced health professionals.

This is the first time I've been paralyzed, so someone has to tell me, ‘you should do this and that….’ They don't just give orders, they also tell you why. However, there are also things that they had to decide for me, such as medication or activities in the therapies….

Looking back several years post‐injury, participants reported that their initial dependency even led to the unconscious surrender of any wish to decide autonomously and the assumption that the therapists and nurses were incharge of everything. In this initial phase, participants experienced questions about their preferences as difficult and disturbing. Trustworthy support by health professionals was essential to enable patients to engage in any kind of decision making. At the beginning, making simple decisions, like choosing what to eat, was valuable in regaining the ability to make decisions. With support from the rehabilitation team, patients were able to begin changing their behaviour.

Decision for the “new life”:

And that was, I believe, the key moment: it was then that I said to myself, ok, now you have to deal with the fact, that you will be in a wheelchair the rest of your life…

Participants perceived the decision for their new life in a wheelchair as one of the most crucial moments during the process of first rehabilitation. With this decision, and their commitment to their new life, patients became more active and engaged. They described this as their first milestone on the long journey of acceptance and building up their own new concept of life.

Increasing decisional ability during inpatient rehabilitation:

Well I think that if you are capable of making an informed decision, then to just handle the whole situation more or less, …you need at least a fortnight…all the consequences, … one needs enough time to think things over.

As their health condition stabilized, patients started to engage actively in the rehabilitation process. They became more aware of their ability to actually engage in the decision‐making process, patients started to feel responsible for their decisions and actions, including the consequences of their decisions.

Decision making during the discharge phase:

At the end of the initial rehabilitation, the participants underlined the importance of decision making in their “new life” and their ability to make decisions independently. This seemed to be a good indicator that they were ready for discharge. Despite the many decisions that had to be made during the discharge phase, patients felt prepared to leave the hospital from the moment they were able to envision their new life at home.

### 3.2 Participants’ physical, psychological and personal factors

Participants reported that their feelings, health condition and physical activities influenced their ability to make decisions.

When I'm having a crappy time, then I don't make any decisions…a therapist once told me that you should simply enjoy life and make decisions later, when you have the energy back again.

…if you aren't physically fit, then at some point you are also not really mentally fit… So, that's somewhat… just, somehow you feel like… you're mentally there, but you are actually mentally somewhere else, I don't really know where.

During the acute phase, the initial shock, the pain and the medication side‐effects reduced their decision‐making skills and the capacity to express their opinion. On the other hand, recovery restored their willingness and ability for decision making and to communicate their opinions.

I think it's often the case, that during the first rehabilitation, perhaps, yes, maybe due to the trauma that you had or you still have, perhaps you're still suffering from, you don't express often enough what you want or don't want.

Only two participants, both having been paralysed for many years, stated explicitly that the SCI induced a psychological shock influencing their ability to make decisions. Stagnation of recovery or loss of physical activity due to temporary illness, such as a pressure ulcer, spasticity or urinary tract infection, was closely linked to their mental and psychological abilities. Correspondingly, regaining physical abilities was linked to mental strengthening.

Your self‐esteem improves, of course, or sort of. If you are able to do things on your own, then, eventually, you may start to speak up for yourself, if you, yes, right, if you don't agree with what is happening…

Patients’ progress as well as setbacks during their rehabilitation, their appearance, their body perception and the image of themselves played an important role in the process of rehabilitation and decision making. Once they were independent in various activities, patients were encouraged to start making personal decisions again. They reported an increasing urge to become as independent as possible and described this feeling as an inner voice, which called them to be active and to engage in their own life. They also described that as time went by, their pre‐SCI, well‐known personal characteristics in making decisions returned, yet at the same time somehow altered by the SCI.

### 3.3 Social and organizational factors

If any decisions had to be made, … I was able to discuss it with everyone, the nurse, the physio, the occupational therapist, the psychiatrist, the doctors… They explained the pros and cons to me… I always had a good fundament for making my decisions… they know best what is needed.

Being aware of their reduced decision‐making skills during the acute phase after an SCI, participants were grateful when being cared for in a respectful way and appreciated that most of the important medical decisions were taken by an experienced therapeutic team rather than by themselves. The participants gave detailed descriptions of the influence the facility had on their decision making. The building with its friendly architecture, the spacious rooms, being surrounded by nature—such as the view of the lake and the mountains—were all mentioned as positive and helpful elements. An additional positive experience they mentioned was contact with visitors from the local community who came to the restaurant and the swimming pool located in the centre of the rehabilitation hospital. The building was described as ideal with enough space, automatic doors and adapted toilets and staffed by friendly and empathetic personnel. It was experienced as a perfect, protected and especially adapted facility different to the real world.

Many factors such as the length of the first rehabilitation and the awareness of a need for lifelong care influenced the relationship between the patient and the hospital staff, leading to varying degrees of experienced personal dependency. Overall, most of the participants were satisfied with the care they received, the friendly and positive atmosphere of the institution even though there were instances when they expressed criticism. The rigid structures and rules of the institution were experienced divergently. Some perceived them as oppressive and restrictive to new ideas, whereas others found them helpful. Only time pressure and stress were unanimously described as negative factors.

One special aspect was raised several times, namely how strongly the institution highlights the image of the “wheelchair user” as being an active and powerful person. Consequently, for some patients this image led to additional uncomfortable pressure when they felt they were not able to achieve this role themselves.

In addition to the influence of the therapeutic team, many patients mentioned the support of peers as an important factor. Peers encouraged and supported their decision‐making ability. Furthermore, meeting peers who were also patients in similar conditions and states of disability made some feel better in comparison with others who were suffering more or who were severely dependent. The immediate family, friends and partners also influenced their feelings significantly. For example, a separation or divorce was mentioned as a negative factor with an immense impact on their life. Many participants also described worrying about their families and relatives ability to cope with their new situation. They felt that the people close to them were not being given sufficient attention and support, although they were also similarly suffering.

The influencing factor time:

The aspect of “time” was mentioned in the context of “time to discuss” or “time to be informed” as well as in the context of the “time line of the rehabilitation process” including the “right moment for discharge” and “time structure in the rehabilitation program” as a whole.

Lack of “time to discuss” and time‐stress, particularly for doctors, had a negative impact on decision making and the interviewees underlined the need to be given sufficient time as part of being taken seriously.

The intensity of the first rehabilitation programme was experienced quite differently among the participants. Some participants mentioned that the programme was so intense and overwhelming that they felt under tremendous pressure. They did not have enough time to reflect on or think about their new life situation. Others, in contrast, spoke for tighter programmes in retrospect, to achieve their goals in a shorter time.

Together with the standard rehabilitation programme including physical and occupational therapy, nursing and therapy with social workers, patients had the opportunity to choose additional therapies. These included psychotherapy, Feldenkrais, a somatic educational therapy to increase kinaesthetic and proprioceptive awareness of functional movement, art or music therapy or activities such as computer or language courses. They were informed about these options by their physicians, therapists or by other patients. Being able to choose between those activities and additional therapies was mentioned as an important part of exercising decision‐making skills and to make the rehabilitation programme more tailored to the individual. These activities gave the patients a sense of empowerment and being capable again.

… the first rehab is at the clinic and the second is at home….

The transition from the hospital to home raised ambiguous feelings. It was a great challenge and required many decisions to be made. Even though participants had experienced the “real world” outside of the rehabilitation centre on several occasions, they wished to have been better prepared for the challenges that lay ahead of them. There was a perceptible difference between discussions and preparations to leave the institution and their actual experiences in the community once discharged in the “real world.” For example, they felt poorly prepared for their restricted participation in society due to environmental barriers or due to the reactions of others. The time it took to take responsibility and to be part of decision making differed from participant to participant, but for all of them the factor “enough time,” was paramount.

Goal setting and time:

Rehabilitation goals were experienced in many different ways. Goal setting was experienced as a major motivation but also led to stress.

…when you realize you will not reach this goal. It is a real frustration.

Mr. X (doctor) said, you will leave this clinic as an independent person. On one hand, this has really motivated me, on the other, it has put an insane amount of pressure on me, because we keep having to extend the rehabilitation period because there are always complications.” … “After a while, I realized that I wouldn't leave the hospital independently…

Patients mentioned goal setting should be a continuous interactive process throughout the rehabilitation period. As it becomes clearer that some goals are unrealistic over time, adaptation of goals might be needed. The interactive process would allow them to become more and more part of this goal‐setting process.

### 3.4 Behaviour and attitude of health professionals

Many times, the doctors and the patient didn't listen to each other. Finally, I said, I feel that I am not taken seriously… they could really say anything to me. But that never happened… who am I really? …the exchange is terrible between doctors, and just enough between doctors and nurses, and worse between doctors and patients. That shouldn't be the case, you want to be taken seriously.

In general, all health professionals such as physicians, nurses, physical and occupational therapists were judged as being experienced as well as kind, friendly, helpful and courteous. In the process of decision making, communication and information played an important role. Some physicians, however, were described as being insensitive, not listening attentively and interrupting patients. Medical information was often missed or not understood. Sometimes the diagnoses in the medical report were different from the diagnoses the patient understood. Participants often lacked the courage to ask physicians for information. There was a discrepancy between the expectation that the patient should be responsible for handling his medication and the information provided by the medical team when the medication regime was changed. When this occurred, participants did not feel respected and taken seriously.

Participants were very interested to get the latest information about medical topics, new medications, possible side‐effects and the like. They also required more information about complex issues such as pain, sexuality and advanced directives. Information was paramount to develop decision‐making ability.

If you are in pain, really in pain, then you are happy about everything that you are told about what you could do about it, aren't you? And I have to deal with the pain, that's clear. I realized that I can't go on living with so much pain. So, you are happy with any details they give you.

Participants wished to have been taken more seriously in their decision making concerning their own rehabilitation process as they felt they knew their own rehabilitation potential best. They suggested a better dialogue with health‐care professionals about the rehabilitation programme. This would allow them, with the guidance of the rehabilitation team, to appropriately integrate their individual ideas into their rehabilitation plans.

Lastly, the health‐care personnel's ability to facilitate and maintain hope was particularly important to participants—even when faced with an uncertain prognosis. Retaining hope played an important role in continuing to be motivated and engaged in the rehabilitation process.

## 4 DISCUSSION

The main findings of this study include the patients’ need to be respected with their capacities and limitations in their decision‐making ability during the rehabilitation programme. In the acute phase, patients feel severely restricted in their decision‐making capacity and are comfortable leaving decisions to the health‐care professionals. The patient's acceptance of beginning a new life with an SCI is a turning point in the acute rehabilitation process. From the patient's point of view, decision making is a rehabilitation goal, which is worth working for.

We learned that a friendly atmosphere is helpful while contradictory information hampers participation in decision making. At the time of their discharge, all patients in our sample felt able to make decisions by themselves, indicating that they regained confidence in their decision making skills over time.

### 4.1 Tools and skills to support decision‐making capacity

This study provides information on how to support the patient's decision‐making ability after a newly acquired SCI, delineating factors which may hinder or facilitate the ability to make decisions from the patient's perspective. As proposed by other studies,^13^, ^21^ our study reinforces that an optimistic, friendly and respectful atmosphere, with opportunities for self‐determination and decision making, is of major importance for the rehabilitation process. Negative factors such as time pressure, rigid structures and poor communication, for example, limit a patient's ability for decision making.^13^, ^19^, ^23^

The factor time was often mentioned in different context. Time was a main influencing parameter on the daily organization and intensity of therapies, the duration of the rehabilitation and the timing of information.^21^, ^29^ Because patients experienced the intensity of the rehabilitation programme in disparate ways, setting up an individualized rehabilitation programme and integrating patients in the goal setting would help.

Goal setting during rehabilitation is an important instrument to support a patient's participation in decision making.^27^, ^30^ Achieving the best level of physical independence and quality of life is most successful when the patient is fully integrated in the goal‐setting process.^23^, ^26^, ^29^, ^31^, ^32^ In our study, patients experienced their occupational therapists to be helpful in the goal‐setting process. These goals were mainly concerned with decision making about auxiliary means, living and working environment. In contrast, our patients perceived physicians in general as being unhelpful in the process of goal setting by being too directive and authoritarian. Indeed, it is known that most physicians are not accustomed to involving patients in the goal‐setting process and see themselves as the experts in their highly specialized field.^33^, ^34^ The process of goal setting is linked to perception about prognoses and retaining hope, as well as the adaptation to and acceptance of realistic goals. It remains a challenge to set realistic goals while knowing that the patient's outcome cannot be predicted with certainty.^29^

### 4.2 Recommendation for shared decision making: patients’ view of health professionals

Patients resented not being informed about changes in medication or planned examinations, about the next steps in rehabilitation and about responsibilities within the team. These findings were similar to those from earlier studies.^17^, ^19^ On the other hand, meeting face‐to‐face with physicians who were dedicated, took time and showed empathy, were mentioned as important positive factors in their rehabilitation. Patients found starting with simple decisions helpful, before moving on to more complex ones. The patients’ willingness was indispensable to becoming as active and as independent as possible. This individual patient motivation together with the health professional's sensitive guidance, partnership, teamwork and a respectful attitude is described to be the ideal atmosphere to support the patient in this process.^21^, ^27^

Information about their prognosis was particularly important to patients during rehabilitation and affected their decision‐making process. Similar to the findings of other studies, the wish to be informed about the prognosis predominates^35^ and honest information after a trauma serves as orientation and supports the self‐healing process.^36^ In the unique situation of a newly acquired SCI, patients in our study with a newly acquired SCI appreciated health‐care staff maintaining hope for recovery (including achieving better‐than‐expected rehabilitation goals), even as they acknowledge uncertainty about the final prognosis. This hope, realistic or not, aided the engagement in the early phase after injury.

### 4.3 Political and ethical background of patient's decision‐making capacity

Autonomy and self‐determination are fundamental rights and values in modern western democratic societies independent of the actual decision‐making capacity of the individual person.^3^, ^10^ Out of respect for these rights, treatment decisions are preferably made according to a patient's preferences and values.^6^

Our findings show that, in completely new life situations, the will of the patient to engage in decision making is limited. The study indicates the patient's decision‐making ability is often restricted at the beginning of the patient's rehabilitation, and however, develops progressively throughout the rehabilitation process.

The political and legal background should provide an individually adapted framework for health‐care professionals, patients and relatives on how to deal with patients in such situations. In our view, health‐care professionals need knowledge about the legal framework, additional education on how to recognize a patient's ability for decision making and an understanding of how to improve a patient's ability for decision making.

### 4.4 Clinical relevance

Awareness of the discrepancy between the right to respect autonomy and the diminished ability for decision making needs to be further developed and guidelines on how to cope with such situations have to be implemented. The medical team and its willingness to respect the patient's right to autonomy and shared decision making, with the ultimate aim to support self‐determined living is a key element in the rehabilitation. It is of major importance that patients are also able to accept the regaining of their decision‐making independence as an important goal in their rehabilitation. Information about the patient's medical condition should be provided in a patient‐centred way. Knowledge about influencing factors and phases in the rehabilitation process after an acute SCI as well as skills to recognize the patient's individual situation concerning his/her decision‐making ability is necessary to improve the rehabilitation process.

Based on the findings in this study, individualized education and information could be helpful with the goal of enabling decision making during the rehabilitation. This should be integrated in the treatment process of a first rehabilitation in addition to the availability of standardized educational materials. The combination of information and patient‐centred care should be also applied in interdisciplinary meetings with patients and their relatives as core elements of the rehabilitation process. Furthermore, medical communication training for all health professionals, supervision for medical doctors and different programmes on conflict management could improve shared decision making.

### 4.5 Limitations

This study included selected participants with SCI, most of whom have stable living conditions, sometimes many years post‐discharge from the initial inpatient rehabilitation and willing to participate in the interview about decision making and autonomy. Therefore, selection bias might have occurred in choosing patients with a high capacity for introspection. For those many years after the onset of SCI, a memory bias influenced by other life events and personal development might have occurred.

Because all participants were recruited out of one rehabilitation clinic, the generalizability, especially in the field of organizational aspects, should be taken with caution. Although the results were in line with the literature in describing the influencing factors in the process of decision making.^13^

The focus of the interview was limited to personal factors. Questions concerning the social and cultural background of the participant were not part of the questionnaire. Consequently, important influencing factors might have been missed.

## 5 CONCLUSION

Patients with SCI, together with their therapeutic team, face a complex health condition with the risk of many complications, and physical and psychological dependencies. This is compounded by the patient's wish to become as independent as possible, to achieve the highest level of autonomy and ultimately the best quality of life.^1^ From the patient's perspective, based on a stable health condition and complex knowledge about the new health situation, the ability for decision making leads to the intended autonomy in the new identity.^20^ Therefore, one of the main goals in the primary SCI rehabilitation, beside achieving medically stable health and physical independence, is to develop the best decision‐making ability in the goal‐setting process.^23^ Supporting decision making starts with the commitment of the health‐care team to respect patients in their process of achieving increased decision‐making ability, to assess the actual capacity for decision making of the patients as part of the treatment diagnoses and to establish a positive atmosphere for the development of the best possible decision‐making skills.

## DISCLOSURES

Financial disclosure statements have been obtained, and no conflict of interests have been reported by the authors or by any individuals in connection with this article.

## CONFLICT OF INTEREST

The authors declare no conflict of interests.
